# Comparative evaluation of apical seal integrity in endodontically treated teeth with and without apicoectomy

**DOI:** 10.6026/973206300213658

**Published:** 2025-10-31

**Authors:** Jalison Jacob Cheruvathoor, Nitin Pratap Shishodia, Bhavya Yadav, Satish Kumar Ram, Amrit Podder, Syed Haider Mehdi

**Affiliations:** 1 Department of Conservative Dentistry and Endodontics, Penang International Dental College, Butterworth, Penang, Malaysia, Asia; 2 Department of Oral Medicine & Radiology, Seema Dental College & Hospital, Rishikesh, Uttarakhand, India; 3 Department of Conservative Dentistry and Endodontics, Faculty of Dental Sciences, PDM University, Bahadurgarh, Haryana, India; 4 Department of Oral and Maxillofacial Surgery, Awadh Dental College & Hospital, Jamshedpur, Jharkhand, India; 5 Department of Physiology, Teerthanker Mahaveer Medical College & Research Centre, Teerthanker Mahaveer University, Moradabad, Uttar Pradesh, India; 6 Department of Conservative Dentistry and Endodontics, Sardar Patel Post Graduate Institute of Dental and Medical Sciences, Lucknow, Uttar Pradesh, India

**Keywords:** Apicoectomy, endodontics, pain, reinfection, seal integrity

## Abstract

The integrity of apical seal and results after treatment of teeth treated endodontically and treated without apicoectomy is of interest. Eighty patients were enrolled in the study, by allocating 40 of them apicoectomy and 40 conventional root canal fill. Data indicated that the apicoectomy-treated group performed much better and experienced fewer post-treatment complications such as pain and reinfection in the absence of apical seal integrity. Data shows that the apicoectomy improves long term treatment success outcomes, particularly when treating a persistent infection. Thus, in endodontic treatment, apicoectomy can be considered a good alternative that can be used to enhance results.

## Background:

Endodontic therapy attempts to clear infections and maintain the natural construct of the tooth through disinfection of the pulp and sealing the root canal system [1]. Nonetheless, in some instances where ordinary root canal treatment is unable to solve the infection, further surgical procedures might be needed [2]. An apicoectomy, a surgical procedure that removes the apical end of the root and some infected tissue, is one of them [3]. The effectiveness of endodontic procedures, whether a surgical or conventional method, is dependent mainly on the integrity of apical seal- the barrier that limits the entry of the microorganism back into the root canal system and initiating another infection [4]. Apical seal is an essential requirement that determines the success of endodontically treated teeth in the long term. During the traditional root canal treatment, filling material is inserted which seals root canals [5]. But sometimes in the condition of chronic infection whereby complications may arise such as apical periodontitis, the apicoectomy is usually conducted to eliminate the infection tissue and assure a sounder seal at the root tip. It is used so that chronic inflammation can be prevented along with ingress of bacteria by inserting a filling material to the root-end, which is usually supplemented by filling the rest of the root structure [6]. The effectiveness of apical seal can be affected by a number of factors including the kind of the sealer, type of the root-end filling material, and the technique adopted by the surgeon [7]. There are materials such as mineral trioxide aggregate (MTA) and resin based materials that are used in apicoectomy and they have diverse abilities of sealing. The effectiveness of various materials used in filling root canal has been studied widely in normal root canal therapy [8]. The data on the comparison of the effectiveness in long-term seal between the teeth involving conventional endodontic therapy and apicoectomy is limited. This research gap poses a serious question mark on the durability and reliability of the apical seal even post an apicoectomy particularly with the influence of variables of postoperative healing, possibility of reinfection and success of the treatment [9]. Therefore, it is of interest to determine the comparative effectiveness of the apical seal in endodontically treated teeth with and without apicoectomy.

## Methodology:

This study is a comparative, prospective clinical trial designed to evaluate the apical seal integrity in endodontically treated teeth with and without apicoectomy. A total of 80 patients will be included in the study, with 40 patients in each group: one group undergoing apicoectomy (Group A) and the other receiving conventional endodontic treatment without apicoectomy (Group B). The inclusion criteria for the study include patients aged between 18 and 60 years with a history of single-rooted teeth undergoing endodontic treatment, those with periapical lesions or persistent infection after initial endodontic therapy, and patients with adequate periodontal health. Patients with systemic conditions affecting wound healing, teeth requiring additional surgical procedures, or those with complicated root canal anatomy will be excluded from the study. In Group A, patients will undergo conventional root canal treatment followed by apicoectomy, where the apical 3-5 mm of the root is resected, and the root-end is filled with a biocompatible material such as mineral trioxide aggregate (MTA). In Group B, patients will undergo conventional root canal treatment without surgical intervention, using gutta-percha or an equivalent material to fill the root canal. Pre-treatment assessment will involve a comprehensive clinical examination and digital radiographs to assess the condition of the tooth and extent of the periapical lesion. Apicoectomy in Group A will be performed using a surgical microscope under local anesthesia to ensure proper removal of infected tissue at the apical region and root-end filling. Post-operative care will include antibiotics, analgesics, and oral hygiene instructions. Follow-up appointments will be scheduled at 1, 3, and 6 months post-treatment to assess healing and check for any signs of reinfection or complications. The primary outcome measure will be the integrity of the apical seal, assessed through radiographic analysis, evaluating periapical radiolucency, root-end filling adaptation, and any voids or gaps. A secondary outcome measure will be the clinical success rate, including assessments for post-treatment pain, swelling, and signs of reinfection. The data will be analyzed using statistical methods such as chi-square tests for categorical variables and independent t-tests for continuous variables, with a significance level set at p<0.05. This methodology will provide a robust comparison of the apical seal integrity between endodontically treated teeth with and without apicoectomy, helping to determine the comparative success of these two treatment modalities. Therefore, this study is important to determine the effectiveness of apicoectomy versus conventional endodontic treatment in terms of apical seal integrity and long-term treatment outcomes.

## Results:

The study included 80 patients, divided equally into two groups: 40 patients in the apicoectomy group (Group A) and 40 patients in the non-apicoectomy group (Group B). The analysis of the gender distribution across both groups showed a relatively balanced representation of males and females. In Group A, there were 22 males (55%) and 18 females (45%). In Group B, there were 24 males (60%) and 16 females (40%). The overall male-to-female ratio for the study population was 46 males (57.5%) and 34 females (42.5%) (Table 1 - see PDF). The mean age of patients in Group A was 42.3 years (SD ± 10.2), while in Group B, the mean age was 41.8 years (SD ± 9.7). The age distribution was similar across both groups, with most patients being within the 30-50 age range. The age difference between the two groups was not statistically significant (p > 0.05) (Table 2 - see PDF). In terms of apical seal integrity, radiographic analysis showed that 90% of teeth in Group A (apicoectomy group) had an excellent or good apical seal, defined as no significant periapical radiolucency and good adaptation of the root-end filling material. In contrast, only 70% of teeth in Group B (non-apicoectomy group) achieved an excellent or good apical seal. The difference between the two groups in terms of apical seal quality was statistically significant (p < 0.05), suggesting that apicoectomy with root-end filling significantly improves apical seal integrity compared to conventional root canal treatment alone (Table 3 - see PDF). Post-treatment complications such as pain, swelling, and signs of reinfection were evaluated during follow-up visits. In Group A, 5 patients (12.5%) reported mild discomfort, which resolved with analgesics, and no signs of reinfection were noted. In Group B, 9 patients (22.5%) reported pain or swelling at the 1-month follow-up, and 2 patients (5%) showed evidence of reinfection, requiring retreatment. The occurrence of post-treatment complications was significantly higher in Group B compared to Group A (p < 0.05) (Table 4 - see PDF). Overall, the results indicate that apicoectomy provides a superior apical seal and reduces the incidence of post-treatment complications compared to conventional root canal treatment. Therefore, the apicoectomy procedure appears to enhance the long-term success of endodontically treated teeth, especially in cases with periapical infection or poor initial healing. The findings support the clinical utility of apicoectomy as an effective intervention for improving the outcomes of endodontic therapy.

## Discussion:

The main purpose of this research was to compare the apical seal integrity and outcome of the post-treatment of the endodontically treated teeth with and without apicoectomy. The findings of this study indicate that apicoectomy could yield extraordinary benefits as it promotes integrity of apical seal level, with a better proportion of good or excellent apical seals reported in apicoectomy group (90%) than non-apicoectomy cohort (70%). In addition, post-operative complications, such as pain, swelling, and reinfection, were much less in the apicoectomy group, which proves once again the positive nature of this particular surgical intervention and its impacts on the overall outcome in the long term. A number of studies were made in the past to study the effect of apicoectomy on integrity of apical seal as well as clinical outcomes. The findings of study conducted by Pushpalatha *et al.* (2022) [10] found that the incidences of reinfection and improvement of apical sealing were observed due to the use of the root-end filling materials like mineral trioxide aggregate (MTA) to fill root ends. On the same note, Lindeboom *et al.* (2005) [11] also noted that superior outcomes were recorded when apicoectomy was done in root-end filling and partial filling of MTA as opposed to filling of conventional root canal. They have described a success rate to be higher and postoperative complications in patients subjected to apicoectomy, and by so doing, they started the notion that apicoectomy provides a stronger approach in treating recurrent apical infection. Conversely, Ajayi *et al.* (2018) [12] compared the use of apicoectomy and retreatment of teeth which had periapical pathology. Although their findings established that both operations were similar in terms of success rate, they observed more cases of postoperative pain and swelling caused by apicoectomy. Such difference can be explained by the sample size of the study, methods of treatment or by incorporation of the cases presenting complicated anatomic problems. The post-operative complications and pain in the apicoectomy group were scarce, as the study implies, and this may have been due to the revised surgery and adequate post-surgery management of the procedure. In a research that was conducted by Kruse *et al.* (2016) [13], the long-term success of root-end filling procedures using MTA in apicoectomy surgeries was appraised and a considerably larger percentage of integrity of apical seal and overall clinical success was observed to far exceed other materials such as gutta-percha. The same is corroborated by the current study, in which the root-end filling materials or MTA was employed to provide superior apical seal. According to their studies, the biocompatibility of MTA, its sealing qualities are more effective to prevent reinfection, which may be the reason that led to the high success rates found by apicoectomy group in the given study. Alternatively, Gulsever *et al.* (2025) [14] also compared apicoectomy and non-surgical retreatment in periapical lesions and reported both procedures to be equally effective with respect to sealing the apical region. Although the study acknowledged that apicoectomy was more beneficial when there was vast root resorption or when there was an unsuccessful root canal treatment caused by complexities in anatomy. Their results reveal that apicoectomy, in some cases, may be very effective, however, some cases of apicoectomy may be unnecessary which is put in context with the current study in which apicoectomy was done on patients whose apical pathology was persistent in spite of the initial root canal treatment.

## Conclusion:

Apicoectomy significantly improves apical seal integrity and reduces post-treatment complications compared to conventional root canal treatment is shown. The findings align with previous research, supporting apicoectomy as an effective intervention for persistent apical infections and complex endodontic cases. Therefore, apicoectomy should be considered a valuable option for enhancing the long-term success of endodontic therapy.
